# The Clinical Significance of Detecting Blood Supply to the Inferior Parathyroid Gland Based on the “Layer of Thymus-Blood Vessel-Inferior Parathyroid Gland” Concept

**DOI:** 10.1155/2022/6556252

**Published:** 2022-04-13

**Authors:** Jian-Biao Wang, Rong Su, Lei Jin, Liang Zhou, Xian-Feng Jiang, Gui-Zhou Xiao, Ye-Yuan Chu, Fei-Bo Li, Yi-Bing Feng, Lei Xie

**Affiliations:** ^1^Department of Head and Neck Surgery, The Affiliated Sir Run Run Shaw Hospital, Zhejiang University School of Medicine, Hangzhou, Zhejiang 310016, China; ^2^Department of Operation Room Nursing, The Affiliated Sir Run Run Shaw Hospital, Zhejiang University School of Medicine, Hangzhou, Zhejiang 310016, China; ^3^Second Department of General Surgery, Zhejiang Putuo Hospital, Zhoushan, Zhejiang 316100, China; ^4^Second Department of General Surgery, Longyou People's Hospital, Quzhou, Zhejiang 324400, China

## Abstract

**Objective:**

How to preserve the inferior parathyroid gland (IPTG) in situ during central neck dissection (CND) is the major concern of thyroid surgeons. The “layer of thymus-blood vessel-IPTG” (TBP layer) concept showed to be effective in preserving IPTG. The objective of this study was to identify the origin and course of blood supply to IPTG (IPBS) within the TBP layer and to take key points of operation during CND.

**Design:**

This is a retrospective control study. *Participants*. Patients who underwent thyroidectomy plus CND using the TBP layer concept and conventional technique between 2017 and 2019 were enrolled. *Measurements*. The origin and course of IPBS in relation to recurrent laryngeal nerve (RLN) and thymus and prevalence of hypoparathyroidism were detected.

**Results:**

A total of 71.3% of IPTGs (251 of 352) were supplied by ITA branches, defined as type A. Type A was further divided into Types A1 (branches of ITA, coursing laterally to the RLN (53.1%, 187 of 352)) and A2 (branches of ITA, traversing medially to the RLN (18.2%, 64 of 352)). Type A2 was more common on the right side than on the left side (*P* < 0.001). Fifty-five (15.6%) IPTG feeding vessels originated from the thymus or mediastinum. Nineteen (5.4%) IPTGs were supplied by branches of the superior thyroid artery. The incidence of transient hypoparathyroidism decreased from 45.7% to 3.6% (*P* < 0.001), in the TBP layer group compared with the conventional technique group.

**Conclusion:**

The origin and course of IPBS follow a definite pattern. This mapping and precautions help surgeons optimize intraoperative manipulations for better preservation of IPBS during CND.

## 1. Introduction

The parathyroid gland is part of the endocrine system and consists of four tiny glands typically found on the back of the thyroid gland. The parathyroid chief cells are responsible for secreting parathyroid hormone, which plays a pivotal role in calcium-phosphate metabolism. In case of inadvertent parathyroid gland injury during total thyroidectomy, postoperative hypocalcemia may occur [1]. Therefore, how to preserve parathyroid glands in situ during thyroid operation is one of the most important concerns of thyroid surgeons.

Concerning parathyroid gland preservation in situ during thyroidectomy, the classical concept of “meticulous capsular dissection” [2] has been followed in the clinic, and the incidence of hypoparathyroidism after thyroidectomy has fallen since then. Therefore, the actual difficulty at present has become inferior parathyroid gland (IPTG) preservation in situ during central neck dissection (CND) in thyroid cancer because IPTGs enjoy a more variable position in the adult neck and are located in the area of the central neck lymph node dissection.

To solve this problem, a new concept of “layer of thymus-blood vessel-inferior parathyroid gland” (a TBP layer) was first put forward by Dr. L. Xie in 2014 [3]. The TBP layer can be explained in two ways. On one hand, the thymus, the IPTG, and blood vessels connecting them are located in one layer. Therefore, the IPTG could be found and preserved without difficulty if this layer is built up. On the other hand, the layer covers the common carotid artery (innominate artery), the trachea, and the area of paratracheal lymph nodes between them, indicating that the IPTG and its layer are not within the scope of paratracheal lymph node dissection. Consequently, the dissection completeness of this area is not discounted even with preserved IPTG in situ. We have applied this concept to perform CND clinically and found an increased rate of IPTG preservation in situ and decreased incidence of transient postoperative hypoparathyroidism [3]. Although it is known that IPTG generally receives branches from the inferior thyroid artery (ITA) [4–6], its blood supply (IPBS) and relationship with the TBP layer have not been clearly described to date. This study aimed to identify IPBS within the TBP layer during CND for thyroid cancer and to discuss the clinical value of such detection.

## 2. Materials and Methods

### 2.1. Study Population

This was a retrospective analysis of patients with papillary thyroid carcinoma (PTC) who underwent thyroidectomy plus ipsilateral or bilateral CND between May 1, 2017, and September 30, 2019, in the Department of Head and Neck Surgery, the Affiliated Sir Run Run Shaw Hospital, Zhejiang University School of Medicine.

Exclusion criteria were previous thyroidectomy and preoperative hyper- or hypoparathyroidism.

Patients who underwent total thyroidectomy plus CND using the TBP layer concept and with the IPBS detected were enrolled in the study group. This group was compared with a control group of patients who underwent total thyroidectomy plus CND with conventional operative techniques during the same period.

The study protocol was approved by the ethics committee of the Affiliated Sir Run Run Shaw Hospital, Zhejiang University School of Medicine. The same surgeon (L. Xie) and an assistant (J. B. Wang) performed all operations in the study group.

A surgeon (G. Z. Xiao) and an assistant (X. F. Jiang) performed the operations in the control group.

### 2.2. Surgical Decision-Making

In accordance with the current American Thyroid Association (ATA) guidelines [7], total thyroidectomy was performed in the patient who meets one of the following criteria: thyroid tumors >4 cm, gross extrathyroidal extension, or clinically apparent metastatic disease to nodes (clinical N1) or distant sites (clinical M1). The indication of CND in PTC patients was in line with the expert consensus of the China Thyroid Association [8]. Ipsilateral CND was performed routinely on the affected side, regardless of whether the central neck lymph nodes were clinically metastatic. Bilateral CND was carried out in patients with tumor(s) located in the isthmus and/or both thyroid lobes or individuals with clinical metastasis in the neck's lymph nodes.

Modified lateral neck dissection (LND), including levels II–IV, was performed only in patients with clinically evident nodal disease in the lateral neck under preoperative ultrasonography, with the ultrasound-guided fine-needle aspiration of the lateral suspicious node showing positive results.

The extent of CND strictly followed the ATA guidelines [9]. Bilateral CND involved the removal of the prelaryngeal, pretracheal, and both the right and left paratracheal nodal basins. Unilateral CND involved the removal of the prelaryngeal, pretracheal, and one paratracheal nodal basins.

All patients underwent direct laryngoscopy preoperatively for the evaluation of vocal cord mobility. An intraoperative neuromonitoring instrument (NIM-Response 3.0 System; Medtronic Xomed, Jacksonville, FL, USA) was used for all patients. Postoperative laryngoscopy was performed only in patients with hoarseness.

### 2.3. The TBP Layer Concept and Its Performance Features [3]

The TBP layer is the layer of the thymus-blood vessel-inferior parathyroid gland, which can be explained in two ways [3]. On one hand, the thymus, the IPTG, and blood vessels connecting them are located in one layer. Therefore, the IPTG could be found and preserved without difficulty if this layer is built up. Actually, this layer is easily found by using the thymus and blood vessel stumps as references. On the other hand, the layer covers the common carotid artery (innominate artery), the trachea, and the area of paratracheal lymph nodes between them, indicating that the IPTG and its layer are not within the scope of paratracheal lymph node dissection. Consequently, the dissection completeness of this area is not discounted even with preserved IPTG in situ.

Different from the traditional method, lateral margin dissection of the paratracheal lymph node area aims to identify and preserve the TBP layer first rather than directly exposing the common carotid artery [3]. The thymus and the branch stumps of inferior thyroid blood vessels are regarded as references, and the fibro-fatty tissue is removed from them. During this process, the TBP layer is slowly identified and lifted, exposing the common carotid artery (innominate artery) beneath it. The medial border of the common carotid artery is dissected down to the prevertebral fascia. The TBP layer and the common carotid artery are retracted laterally, while the trachea is retracted medially, and the paratracheal compartment is exposed. Next, the envelope of paratracheal lymph nodes is excised with recurrent laryngeal nerve (RLN) protection as in the traditional method [10].

Although it is helpful in meticulous operations, the surgical loupe was not used in this procedure.

### 2.4. Tracing and Classifying the IPBS within the TBP Layer

Best efforts were made to trace the origin and course of blood supply to IPTGs during the thyroidectomy and CND procedures in patients using the TBP layer concept. Thyroid lobectomy was performed strictly according to the “meticulous capsular dissection” concept [2]. The parathyroid glands were not routinely and deliberately exposed during thyroidectomy. In case an IPTG was encountered during thyroid lobectomy, the origin and course of its feeding vessel were detected and preserved. CND was performed according to the TBP layer concept, as described above. It was necessary to determine whether the blood vessel connecting with the TBP layer originated from the deep branches of the ITA before sectioning. After CND, the position and blood supply of the IPTG associated with the TBP layer were confirmed. In case the IPTG was vascularized, its feeding blood vessel was identified and classified according to its relationship with the RLN and thymus.

The IPBS was determined by the fine-needle pricking test. The IPTG was identified and pricked using a 25 G injection needle after CND. A gland with blood oozing after the test was considered to indicate vascularization; otherwise, it was regarded as devascularization. Any devascularized parathyroid gland was removed and autotransplanted into the sternocleidomastoid muscle. The origins and courses of the feeding vessels were further explored and analyzed only for the vascularized IPTGs (within the TBP layer).

### 2.5. Clinical Parameters

The recorded parameters were sex, age, voice change, maximum tumor size, multifocality, extrathyroidal extension, presence of cN1, number of harvested and metastatic lymph nodes, number of IPTGs inadvertently excised, preoperative and postoperative serum calcium, and intact parathyroid hormone (iPTH) levels (available for patients administered total thyroidectomy). The number of inadvertently excised parathyroid glands was verified by examining paraffin-embedded specimens of thyroid lobes as well as the fibrofatty tissue of the central compartment.

The status of IPTGs after CND was determined perioperatively by the surgeon and the assistant, and IPTGs were classified into four categories: C1, preservation in situ (vascularization); C2, autotransplantation (devascularization); C3, removal owing to infiltration by the tumor; and C4, not identified. Blood supply was determined as described above.

### 2.6. Laboratory Assays

Serum calcium and iPTH levels were assessed in patients who received total thyroidectomy before the surgery and 6 a.m. every day after operation until discharge. Most patients were discharged on day 3, depending on the volume of drainage fluid. Serum iPTH levels were measured on a Roche Cobas E601 instrument (Hitachi High-Technologies, Tokyo, Japan; normal range 15–65 ng/l). Serum calcium levels were measured on an Abbott Aeroset Automated Instrument Analyzer (Toshiba Medical Systems, Tochigi-ken, Japan; normal range 2.11–2.52 mmol/l). Ionized calcium amounts were not determined separately in this study.

### 2.7. Definition and Treatment of Hypoparathyroidism

Transient hypoparathyroidism was defined as an iPTH level below the normal range (15 ng/l) after surgery [11–13]. Hypocalcemia was defined as a serum calcium level less than 2 mmol/l (8 mg/dl) or requirement for calcium supplementation for treating clinical symptoms of hypocalcemia, such as distal digital paresthesia or tetany, during the hospital stay [14, 15]. Permanent hypoparathyroidism was defined as subnormal serum iPTH levels, calcium levels below 2 mmol/L, or requirement for calcium and/or vitamin D supplementation for treating hypocalcemia-related symptoms for more than 6 months. In our study, patients who underwent thyroid lobectomy were not included in the analysis of hypoparathyroidism. Oral calcium supplements containing 600 mg calcium plus 125 unit vitamin D3 were used twice daily to treat symptomatic hypocalcemia. Calcium gluconate injection was prescribed for persistent symptomatic hypocalcemia after oral calcium treatment.

### 2.8. Statistical Analysis

Continuous data were presented as mean (standard deviation) or median (range). Baseline patient characteristics, incidence of hypoparathyroidism, proportion of IPTGs preserved in situ, and calcium and iPTH levels were compared between the study and control groups using Student's *t*-test or Mann–Whitney *U* test for continuous variables and Pearson *χ*^2^ test for categorical variables. *P* < 0.050 was considered statistically significant (two-tailed). The data were analyzed with SPSS version 16.0 (IBM, NY, USA).

## 3. Results

### 3.1. Patient Characteristics

A total of 374 patients with primary PTC who underwent thyroidectomy plus CND between May 1, 2017, and September 30, 2019, were included in the present study. A total of 258 patients were treated using the TBP layer concept and with the IPBS detected, while 116 patients were treated with conventional operative techniques. Patient characteristics are shown in Table 1.

### 3.2. Origins and Courses of Blood Supply to IPTGs

The 258 patients treated using the TBP layer concept combined with IPBS detection included 57 men and 201 women, with a mean age of 42.1 years (range, 12–71 years) at the time of the first diagnosis, with concomitant Hashimoto's thyroiditis found in 44 individuals. The characteristics of these patients are listed in Table 1. Overall, 5 different operative procedures were performed: (1) lobectomy (including isthmectomy and pyramidal lobectomy) plus ipsilateral CND (right side, 74 patients and left side, 74 patients), (2) total thyroidectomy plus ipsilateral CND (right side, 8 patients and left side, 8 patients), (3) total thyroidectomy plus bilateral CND (52 patients), (4) total thyroidectomy plus bilateral CND and ipsilateral LND (right side, 19 patients and left side, 18 patients), and (5) total thyroidectomy plus bilateral CND and LND (5 patients). A total of 352 IPTGs were expected to be recovered after 352 unilateral CND (176 for each side). The rates of IPTG preservation in situ were 91.5% (161 of 176) on the left side and 93.2% (164 of 176) on the right side, with no significant difference (*P*=0.548; Table S1 supplementary information).

**Figure 1:**
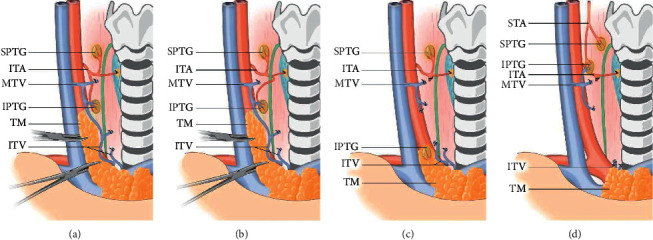
Diagram of the origin and course of blood supply to the inferior parathyroid gland (IPTG).

Of the 352 expected IPTGs, 325 IPTGs with good blood supply were preserved in situ after CND, and the origins and courses of their blood vessels were analyzed. There were three types of IPBS origins:

Type A originated from branches of the ITA. In this study, 71.3% of IPTGs (251 of 352, including 114 glands on the left side and 137 glands on the right sides, respectively) were found to be supplied by branches of the ITA. According to the relationship of ranches of the ITA supporting the IPTG and RLNs, Type A was further divided into Type A1 and Type A2: the IPTG branch of the ITA behind the common carotid artery ascending laterally to the RLN and running into the IPTG was considered Type A1 (Figure 1(a); Figure S1 A1 supplementary information); meanwhile, the IPTG branch ascending medially to the RLN reflected Type A2 (Figure 1(b); Figure S1 A2 supplementary information).

In this study, Type A1 showed no statistically significant difference in terms of side (*P* = 0.455). The frequencies of occurrence were 55.1% (97 of 176) on the left side and 51.1% (90 of 176) on the right one (Table 2). Meanwhile, Type A2 vessels were found in 64 of 325 IPTGs and showed a higher number on the right side compared with the left side (*P* < 0.001). The incidence rates of Type A2 were 26.7% (47 of 176) and 9.7% (17 of 176) on the right and left sides, respectively (Table 2).

Type B originated from the thymic or mediastinal blood supply or within the thymus. Fifty-five IPTGs were uncovered in proximity to or within the thymic tongue, which is a distinct structure at the thoracic inlet, extending from the low thyroid pole to the mediastinal thymus (Figure 1(c); Figure S1 B supplementary information). The incidence rates of Type B vessels were 19.3% (34 of 176) and 11.9% (21 of 176) on the left and right sides, respectively (Table 2). There was no significant difference between the two sides (*P* = 0.056).

Type C originated from the anterolateral branch of the superior thyroid artery (Figure 1(d); Figure S1 C supplementary information). There were nineteen Type C IPTGs (19 of 352, 5.4%), including 13 (7.4%) and 6 (3.4%) on the left and right sides, respectively.

Due to failed in situ preservation, twenty-seven IPTGs (7.7%) were not further analyzed. Four glands were grossly encroached by the tumor and excised together with the tumor. Four glands were excised and autotransplanted into the sternocleidomastoid muscle because their blood supply vessels were infiltrated by the tumor or metastatic lymph nodes. Two glands were likely to receive blood supply from direct branches of the thyroid gland. Five glands were devascularized in the TBP layer and were autotransplanted into the sternocleidomastoid muscle. The remaining twelve IPTGs were not found during the procedures, of which one right side gland was within the thyroid discovered by examination of paraffin-embedded specimens of the thyroid lobe.

### 3.3. The Effect of TBP Layer Concept Combined with IPBS Preservation

Among the 258 patients treated using the TBP layer concept combined with the IPBS detection, 110 patients received total thyroidectomy plus CND who were enrolled in the study group. The control group included sex- and age-matched 116 patients who underwent conventional total thyroidectomy plus CND during the same period. Two group patient characteristics are shown in Table 3. Age, sex ratio, type of CND, Hashimoto's thyroiditis, retrieved lymph node in the central compartment, and vocal cord palsy had no significant difference between the two groups (Table 3). Patients in the study group have larger tumor size, higher rates of cN1, extrathyroidal extension, and central neck lymph node metastases; and more metastatic lymph nodes in the central compartment than patients in the control group (Table 3).

The rates of IPTG preservation in situ were significantly higher in the study group compared with the control group (left side: 91.2% (93 of 102) vs. 45.1% (46 of 102), *P* < 0.001; right side: 92.2% (94/102) vs. 35.8% (39 of 109), *P* < 0.001). The rate of inadvertent excision of the parathyroid gland was also significantly lower in the study group (4.5% vs. 18.1%, respectively; Table 3). Preoperative serum iPTH and calcium levels did not differ between the two groups. However, at day 1 to day 3 after the operation, iPTH and calcium levels in the study group were all significantly higher than those in the control group (Table 4). In the study group, 4 of 110 patients (3.6%) developed transient hypoparathyroidism, compared with 53 of 116 patients (45.7%) in the control group (*P* < 0.001). Permanent hypoparathyroidism occurred in three patients in the control group and none in the study group (*P* = 0.090).

## 4. Discussion

Currently, IPTG preservation in situ during CND remains the biggest challenge for thyroid surgeons. Previous methods are recommended to identify and preserve the IPTG. For example, IPTG is superficial to RLN's coronal plane [16], or the triangular region is not dissected in order to protect the laterally based blood supply of the IPTG [17]. However, it is actually not easy to practice. The proposal of the TBP layer concept makes IPTG preservation in situ simple and efficient, as confirmed in our previous report [3]. In this study, we had more satisfactory results of TBP layer concept combined with IPBS preservation, with success rates of IPTGs preservation in situ of 91.5% on the left side and 93.2% on the right side; the median number of removed lymph nodes in the central neck compartment was 11. The incidence of transient hypoparathyroidism was only 3.6%, significantly lower than patients in the control group, and no patient developed permanent hypoparathyroidism.

Keeping the blood supply well is the key for IPTG preservation in situ. Multiple studies assessing parathyroid blood supply have been performed for more than 100 years, and most thyroid surgeons generally believe that the parathyroid gland could be preserved very well if its blood supply status is completely understood. Theoretically, this is absolutely correct, but clinical practice suggests otherwise because directly searching for the IPBS has the same difficulty as or even more than looking for the IPTG itself during CND. Therefore, the practical value of IPBS research must be greatly reduced without an effective theory or concept to guide IPTG protection. Excitedly, for parathyroid gland preservation in situ, the TBP layer is a very effective concept, which has been confirmed again in this study. Therefore, based on the TBP layer concept followed during CND, the IPBS needs to be recognized and analyzed again for good preservation in situ. In this study, we took the necessary precautions during the operation depending on different IPBS types (Table S2 supplementary information).

Corroborating previous studies, we found three blood supply sources for the IPTG, including branches of the ITA, the thymus or the mediastinum, and the anterolateral branch of the superior thyroid artery, namely, Type A, Type B, and Type C, respectively. More than 70% of Type A IPTGs were identified in this study, in accordance with previous findings [4–6]. We further divided Type A into Types A1 and A2 according to the relationship of branches of the ITA supporting the IPTG and the RLNs. In Type A1, the IPTG branch of the ITA behind the common carotid artery ascended laterally to the RLN and ran into the IPTG; meanwhile, the IPTG branch ascended medially to the RLN, ran across the nerve surface, and entered the TBP layer (IPTG) in Type A2. For Type A IPTGs, special considerations should be given during CND following the TBP layer concept as described below.

During lateral margin dissection of the paratracheal lymph node area and TBP layer building-up, blood vessels near the layer should be considered. They should be preserved if parallel to the long axis of the thymus lobe or common carotid artery. Conversely, they should be cut off if perpendicular to the long axis of the thymus lobe or common carotid artery, running medially and superficially towards the trachea. However, if the blood vessels run from the deep and entered the TBP layer, they probably originate from the ITA and supply blood to the IPTG in the TBP layer. Therefore, they should be preserved (Figure S2 supplementary information).

In order to expose the RLN during the dissection of the paratracheal region, many thyroid surgeons are accustomed to directly incising the fibrofatty tissue from the surface of the RLN. As to Type A2, this is a dangerous manipulation because the IPTG blood supply across the nerve surface must be injured. Therefore, we recommend that the trend of blood vessels across the nerve surface should be identified before incision during the above procedure.

In order to expose the paratracheal region well, thyroid surgeons generally retract the trachea medially and the common carotid artery laterally during CND. However, in the case of Type A2, exposure of this subregion, which is between the cricoid cartilage and the ITA trunk, may be limited in the above manipulation. This is because the RLN traverses the ITA branches and the common carotid artery and limits the lateral retraction of the TBP layer and the carotid artery. For dissection completeness and IPBS preservation, paratracheal dissection can be divided into two parts according to the level of the IPBS, that is, cranial (between the cricoid cartilage and the IPBS) and caudal (between the innominate artery and the IPBS) to the IPBS.

Actually, such subregional dissection is challenging because the superior parathyroid gland and its blood supply from the ITA and the RLN are in this area. Although some surgeons suggest that performing the dissection inferiorly from the trunk of the ITA could achieve an equal completion of dissection in terms of safety [19, 20], because metastatic lymph nodes are rarely found above the ITA trunk, recurrence can be observed in this subregion between the cricoid cartilage and the ITA trunk. Therefore, dissection in this subregion (between the cricoid cartilage and the ITA level) should be emphasized, especially when the TBP layer concept is applied for Type A2 vessels. In addition, the usage of carbon nanoparticles (as a lymphatic tracer) can facilitate this procedure.

In this study, 55 (15.6%) Type B IPTGs were uncovered receiving blood supply from the thymus or the mediastinum. These glands were in proximity to or within the thymic tongue without arterial blood supply originating from the neck. This finding was in accordance with a previous study by Di Marino et al., reporting 15.5% IPTGs are situated in the cornua of the thymus or its immediate vicinity, with 2% of glands included in the thymus parenchyma [21]. Arterial supply to this type of IPTG is generally derived from the internal mammary artery [22], the brachiocephalic trunk, or the aortic arch [21]. This type of IPTG can be easily preserved in situ with the application of the TBP layer concept. When the TBP layer is identified, the gland and its blood supply can be retracted laterally and easily included in the layer.

As for Type C vessels, 19 glands (5.4%) were supplied by branches of the superior thyroid artery (7.4% and 3.4% on the left and right sides, respectively). This finding was in accordance with data reported by Burger et al. suggesting that 11.8% and 2.3% of the left and right IPTGs, respectively, are exclusively supplied by the superior thyroid artery [23]. The superior thyroid artery is usually divided into three branches that embrace the superior thyroid pole, including two located anteriorly and one that runs dorsally to the thyroid superior pole [24]. In this type, the IPTG is supplied by the anterolateral branch of the superior thyroid artery, which may also have branches anastomosing with ITA branches. In this case, the IPBS arising from the superior thyroid artery always travel caudally, runs close to the thyroid parenchyma, and often has short branches connecting to the thyroid parenchyma before entering the parathyroid gland. Delattre et al. found that ITA was absent in 4.5% of cases, in whom the IPTG received its blood supply from a branch of the superior thyroid artery [25]. Delattre et al. also reported that the branch of the superior thyroid artery supply the IPTG is coursing anteriorly to the thyroid gland in some cases and particularly at risk during lobectomy [25]. To keep this type of IPBS intact, the “meticulous capsular dissection” concept should be followed during lobectomy, ligating the individual branches close to the thyroid gland as much as possible. Fortunately, when this type of IPBS is preserved during lobectomy, the IPTG and its blood supply can easily be retracted superiorly and laterally before CND and are not disturbed by the subsequent CND procedure.

It is very important for thyroid surgeons to evaluate whether the IPTG preserved in situ after CND is “live” (vascularized) or not because “dead” IPTG (without blood supply) has much greater odds of resurrection after autotransplantation than that remaining in situ. Presently, there are some ways to evaluate the blood supply of the parathyroid gland, for example, observing the gland's color and plumpness and using the indocyanine green enhanced fluorescence technique [12]. The former is simple but not reliable, while the latter is reliable but needs special expensive equipment. We strongly recommend the fine-needle pricking test, as a simple and reliable tool for evaluating the blood supply of the parathyroid gland. As described in the Methods section, the IPTG was considered to be live with fresh blood oozing from a mini-wound when the parathyroid gland was pricked or its surface scratched with a needle.

This study had some limitations. The status of identified blood supply of the IPTG during surgical operation might be different from that observed in the anatomic dissection of cadavers, considering operative field exposure, the influence of the thyroid disease itself, and so on. For example, we did not demonstrate that IPTG arteries originate from the anastomosing channel between the inferior and superior thyroid vessels, which has been mentioned in previous articles. Although we could identify the IPTG with our rich experience, we did not determine whether there is a supernumerary one. In addition, a small part of IPTGs or their blood supplies were not identified in this study. At last, another limitation has to be pointed out. It is well-known that the levels of postoperative calcium and iPTH are related to all four parathyroid glands, but the state of the bilateral superior parathyroid glands (SPTG) and the IPTG on non-CND side cannot be assessed accurately in this study. The reason is that the SPTGs and the IPTGs on non-CND side do not need to be identified and evaluated routinely if they are not encountered during thyroid lobectomy. However, the authors think the state of the SPTG and the IPTG on non-CND side should be considered similar between the control and study groups after total thyroidectomy because all thyroidectomies are performed by senior operators strictly following “meticulous capsular dissection.” The surgeons included in this study are all senior operators in the same hospital, who have more than 10 years of clinical experience in thyroid surgery and performing more than 500 cases of thyroid operations each year. Furthermore, they perform thyroidectomies strictly following “meticulous capsular dissection.” So we think the difference between the control and study groups caused by the factor of the surgeon is minor in our study.

In conclusion, the origin and course of IPTG's blood supply follow a definite pattern. The favorite types of IPBS are, in the order of frequency, branch of the ITA lateral to the RLN, branch of the ITA medial to the RLN, origin from the thymus or the mediastinum, and branch of the superior thyroid artery. This mapping could help thyroid surgeons optimize intraoperative manipulations for better preservation of IPTG's blood supply during CND following the TBP layer concept.

## Figures and Tables

**Table 1:** Patient demographics.

Parameter	Overall patients	Patients treated with TBP
	(*n* = 374)	Layer concept (*n* = 258)
Age (year)^*∗*^	43.1 (12.1)	42.1 (12.6)
Sex ratio (F:M)	289:85	201:57
Tumor size on histology (cm)†	0.80 (0.20–4.70)	0.90 (0.20–4.70)
Multifocal lesion	214 (57.2)	105 (40.7)
Type of CND		
Unilateral	186 (49.7)	164 (63.6)
Bilateral	188 (50.3)	94 (36.4)
Hashimoto's thyroiditis	84 (22.5)	44 (17.1)
Overall lymph node yield in CND†		
Retrieved	11.0 (1.0–37.0)	11.0 (1.0–37.0)
Metastatic	1.0 (0–25.0)	1.0 (0–25.0)
Central neck lymph node	211 (55.6)	151 (58.5)
Metastases		
Parathyroid excised inadvertently	32 (8.6)	11 (4.3)
Vocal cord palsy	9 (2.4)	3 (1.2)

Values in parentheses are percentages unless otherwise indicated; values are ^*∗*^mean (standard deviation) and †median (range). TBP layer, layer of thymus-blood vessel-inferior parathyroid gland. CND, central neck dissection.

**Table 2:** Origins and courses of blood supply to IPTGs.

Types	Origins and courses	Total number of CND (*n* = 352)	CND in the left side (*n* = 176)	CND in the right side (*n* = 176)	*P* ^*∗*^
A1	Branch of the ITA, lateral to the RLN	187 (53.1)	97 (55.1)	90 (51.1)	0.455
A2	Branch of the ITA, medial to the RLN	64 (18.2)	17 (9.7)	47 (26.7)	<0.001
B	From the thymus or the mediastinum	55 (15.6)	34 (19.3)	21 (11.9)	0.056
C	Branch of the STA	19 (5.4)	13 (7.4)	6 (3.4)	0.099
	Unclear state	27 (7.7)	15 (8.5)	12 (6.8)	0.548

Values in parentheses are percentages. IPTG, inferior parathyroid gland. CND, central neck dissection. ^*∗*^Pearson's *χ*^2^ test. Type A1, the IPTG branch of the inferior thyroid artery (ITA) behind the common carotid artery ascended laterally to the recurrent laryngeal nerve (RLN). Type A2, the IPTG branch of the ITA ascended medially to the RLN. Type B, from the thymic or mediastinal blood supply. Type C, from the anterolateral branch of the superior thyroid artery (STA).

**Table 3:** Baseline characteristics and surgical results for patients who underwent total thyroidectomy and central neck dissection.

	Study group	Control group	*P*‡
	(*n* = 110)	(*n* = 116)	
Age (years)^*∗*^	43.2 (14.0)	45.4 (10.5)	0.200§
Sex ratio (F:M)	90:20	88:28	0.274
Tumour size on histology (cm)†	1.00 (0.20–4.20)	0.80 (0.20–4.50)	0.039¶
Multifocal disease	72 (65.5)	109 (94.0)	<0.001
Extrathyroidal extension	47 (42.7)	28 (24.1)	0.022
Presence of cN1	51 (46.4)	23 (19.8)	<0.001
Type of CND			
Unilateral	16 (14.5)	22 (19.0)	0.375
Bilateral	94 (85.5)	94 (81.0)	
Hashimoto's thyroiditis	31 (28.2)	40 (34.5)	0.308
Overall lymph node yield			
Retrieved^*∗*^	15.6 (7.1)	11.4 (6.1)	0.186§
Metastatic†	2.0 (0.0–25.0)	0.0 (0.0–16.0)	<0.001¶
Central neck lymph node metastases	78 (70.9)	57 (49.1)	0.001
Parathyroid excised inadvertently	5 (4.5)	21 (18.1)	0.001
Vocal cord palsy	3 (2.7)	6 (5.2)	0.347

Values in parentheses are percentages unless indicated otherwise. Values are ^*∗*^mean (standard deviation) and †median (range). CND, central neck dissection. ‡Pearson *χ*^2^ test, except §Student's *t*-test and ¶Mann–Whitney *U* test.

**Table 4:** Serum intact parathyroid hormone and calcium levels in patients who underwent total thyroidectomy and central neck dissection.

	Calcium (mmol/l)		*P*†	iPTH (ng/l)		*P*†
	Study group	Control group		Study group	Control group	
Before operation	2.39 (0.11)	2.41 (0.09)	0.129	45.42 (13.63)	45.96 (17.31)	0.801
After operation						
Day 1	2.10 (0.11)	1.98 (0.15)	<0.001	41.63 (15.57)	18.67 (13.38)	<0.001
Day 2	2.11 (0.14)	1.92 (0.18)	<0.001	42.82 (15.66)	18.18 (14.46)	<0.001
Day 3	2.15 (0.15)	1.93 (0.22)	<0.001	41.29	11.41	<0.001§
				(11.22–125.20)^*∗*^	(1.90–44.54)^*∗*^	

Values are means (standard deviation) unless indicated otherwise; ^*∗*^values are median (range). iPTH, intact parathyroid hormone. †Student's *t*-test, except §Mann–Whitney *U* test.

## Data Availability

The data of this study are available in the following link: https://figshare.com/s/e212c9fe32b23c5f2472

## Supplementary Materials

Supplementary Materials Intraoperative views of the origin and course of blood supply to the inferior parathyroid gland (IPTG). (A1) The blood supply of the IPTG arising from the inferior thyroid artery (ITA) traverses laterally to the recurrent laryngeal nerve (RLN). (A2) The blood supply of the IPTG originating from the ITA traverses medially to the RLN. (B) The IPTG received feeding vessels from the thymus or the mediastinum. (C) The IPTG supplied by the superior thyroid artery (Fig. S1 supplementary information). The blood vessels run from the deep and entered the TBP layer; they probably originate from the ITA and supply blood to the IPTG in the TBP layer. Therefore, they should be preserved (Fig. S2 supplementary information). The rates of IPTG preservation in situ were 91.5% (161 of 176) on the left side and 93.2% (164 of 176) on the right side, with no significant difference (*P* = 0.548; Table S1 supplementary information). In this study, we took the necessary precautions during the operation depending on different IPBS types (Table S2 supplementary information).
