# Supplementary material for: The Clinical Significance of Detecting Blood Supply to the Inferior Parathyroid Gland Based on the “Layer of Thymus-Blood Vessel-Inferior Parathyroid Gland” Concept
Source: Int J Endocrinol. 2022 Apr 13;2022:6556252. doi: 10.1155/2022/6556252 (PMC9020968; doi:10.1155/2022/6556252)
Supplement: Supplementary Materials — Intraoperative views of the origin and course of blood supply to the inferior parathyroid gland (IPTG). (A1) The blood supply of the IPTG arising from the inferior thyroid artery (ITA) traverses laterally to the recurrent laryngeal nerve (RLN). (A2) The blood supply of the IPTG originating from the ITA traverses medially to the RLN. (B) The IPTG received feeding vessels from the thymus or the mediastinum. (C) The IPTG supplied by the superior thyroid artery (Fig. S1 supplementary information). The blood vessels run from the deep and entered the TBP layer; they probably originate from the ITA and supply blood to the IPTG in the TBP layer. Therefore, they should be preserved (Fig. S2 supplementary information). The rates of IPTG preservation in situ were 91.5% (161 of 176) on the left side and 93.2% (164 of 176) on the right side, with no significant difference (P = 0.548; Table S1 supplementary information). In this study, we took the necessary precautions during the operation depending on different IPBS types (Table S2 supplementary information). [file 6556252.f1.docx]

**Table S2. Summary of clinical key points of the operation according to IPBS types during CND following the TBP layer concept**

| IPBS types | Usage of  operative concepts* | Key points of the operation |
| --- | --- | --- |
| A1 | Both | Preserve blood vessels parallel to the common carotid artery and running from the deep during TBP layer build-up |
| A2 | Both | 1 Preserve blood vessels parallel to the common carotid artery and running from the deep during TBP layer build-up  2 Don’t directly incise the fibrofatty tissue from the surface of the recurrent laryngeal nerve  3 Further emphasize dissection in the sub-region between the cricoid cartilage and the inferior thyroid artery |
| B | Both | Protect the thymic lobe and thyrothymic horn |
| C | Meticulous capsular dissection | Ligate the individual branches close to the thyroid gland as much as possible, and dissect the inferior parathyroid gland with the vascular pedicle free from the thyroid surface |

*During total thyroidectomy and central neck dissection (CND) for papillary thyroid carcinoma, two operation concepts have been recommended in order to preserve the inferior parathyroid gland (IPTG) *in situ* ^[18]^: (1) “meticulous capsular dissection” in thyroid lobectomy; (2) the TBP layer concept in CND. IPBS, blood supply to the IPTG. CND, central neck dissection. TBP layer, layer of thymus–blood vessel–inferior parathyroid gland.
